# The Mitochondrial Genomes of the Early Land Plants *Treubia lacunosa* and *Anomodon rugelii:* Dynamic and Conservative Evolution

**DOI:** 10.1371/journal.pone.0025836

**Published:** 2011-10-05

**Authors:** Yang Liu, Jia-Yu Xue, Bin Wang, Libo Li, Yin-Long Qiu

**Affiliations:** 1 Department of Ecology and Evolutionary Biology, University of Michigan, Ann Arbor, Michigan, United States of America; 2 School of Life Sciences, Nanjing University, Nanjing, Jiangsu, People’s Republic of China; Rutgers University, United States of America

## Abstract

Early land plant mitochondrial genomes captured important changes of mitochondrial genome evolution when plants colonized land. The chondromes of seed plants show several derived characteristics, e.g., large genome size variation, rapid intra-genomic rearrangement, abundant introns, and highly variable levels of RNA editing. On the other hand, the chondromes of charophytic algae are still largely ancestral in these aspects, resembling those of early eukaryotes. When the transition happened has been a long-standing question in studies of mitochondrial genome evolution. Here we report complete mitochondrial genome sequences from an early-diverging liverwort, *Treubia lacunosa,* and a late-evolving moss, *Anomodon rugelii*. The two genomes, 151,983 and 104,239 base pairs in size respectively, contain standard sets of protein coding genes for respiration and protein synthesis, as well as nearly full sets of rRNA and tRNA genes found in the chondromes of the liverworts *Marchantia polymorpha* and *Pleurozia purpurea* and the moss *Physcomitrella patens*. The gene orders of these two chondromes are identical to those of the other liverworts and moss. Their intron contents, with all *cis*-spliced group I or group II introns, are also similar to those in the previously sequenced liverwort and moss chondromes. These five chondromes plus the two from the hornworts *Phaeoceros laevis* and *Megaceros aenigmaticus* for the first time allowed comprehensive comparative analyses of structure and organization of mitochondrial genomes both within and across the three major lineages of bryophytes. These analyses led to the conclusion that the mitochondrial genome experienced dynamic evolution in genome size, gene content, intron acquisition, gene order, and RNA editing during the origins of land plants and their major clades. However, evolution of this organellar genome has remained rather conservative since the origin and initial radiation of early land plants, except within vascular plants.

## Introduction

Among major lineages of eukaryotes, land plants (embryophytes) have mitochondrial genomes that exhibit several derived features setting them apart from chondromes of other eukaryotes: large and highly variable genome sizes, frequent intra-genomic rearrangements, rich and varied intron contents, highly variable RNA editing levels, and incorporation of foreign DNAs [1], [2], [3], [4], [5], [6]. When and how these evolutionary novelties arose in land plants, which represent a clade spanning at least 475 million years of evolution [7], have remained largely unknown until recently. Over the last twenty years, chondromes from representatives of all major lineages of early land plants and some charophytic algae have been sequenced. These include four charophytes (*Mesostigma viride* [8], *Chlorokybus atmophyticus* [9], *Chaetosphaeridium globosum* [10], and *Chara vulgaris* [11]), two liverworts (*Marchantia polymorpha* [12] and *Pleurozia purpurea* [13]), one moss (*Physcomitrella patens* [14]), two hornworts (*Megaceros aenigmaticus* [15] (despite its change to *Nothoceros aenigmaticus* [16], we will use the original name to be consistent with literature) and *Phaeoceros laevis* [17]), and two lycophytes (*Isoetes engelmanii* [18] and *Selaginella moellendorffii* [19]). These data show that the mitochondrial genomes experienced dynamic evolution during the origin and early evolution of land plants but have remained rather stable within liverworts and hornworts [6]. Further, these data and those from seed plant chondromes [20], [21], [22], [23], [24], [25], [26], [27], [28], [29], [30] suggest that the extremely fluid mitochondrial genomes first found in flowering plants [31], [32], [33] are restricted to vascular plants.

More specifically, these sequenced chondromes indicate that the mitochondrial genome size increased significantly during the origin of land plants and had remained relatively constant in bryophytes, which represent the first stage of land plant evolution [34], [35]. The wide genome size variation is seen so far mostly in angiosperms [26]. Frequent intra-genomic rearrangements did not occur until the emergence of vascular plants. Intron contents, while being relatively stable within most of the major lineages of land plants (liverworts, hornworts, and vascular plants), differ significantly among these lineages. RNA editing clearly occurs in liverworts, the most basal lineage of land plants [34], [36], [37], but only two lycophytes, *Isoetes* and *Selaginella,* have been found to show extremely high levels of editing so far [19], [38]. Finally, no foreign DNA has been seen in any of the bryophyte chondromes, and the first land plant that has sequences of both chloroplast and nuclear origins in the chondrome is the lycophyte *Isoetes* [18].

To determine the mode of mitochondrial genome evolution across the entire diversity of bryophytes, we sequenced chondromes of the moss *Anomodon rugelii* and the liverwort *Treubia lacunosa*. *Anomodon rugelii* is the second moss, after *Physcomitrella patens*, that is sequenced for the chondrome. The two species span much of the clade true mosses, which represents 94% of the moss species diversity [39], [40], [41]. *Treubia lacunosa* represents one of the two liverwort families (Treubiaceae and Haplomitraceae) in the clade that is sister to all other liverworts [40], [42]. Hence, these two chondromes from critically positioned taxa add significantly to the data that already exist and allow a comprehensive examination of mitochondrial genome organization and evolution in early land plants.

## Results and Discussion

### General Features of the *Treubia* and *Anomodon* Mitochondrial Genomes

**Figure 1:**
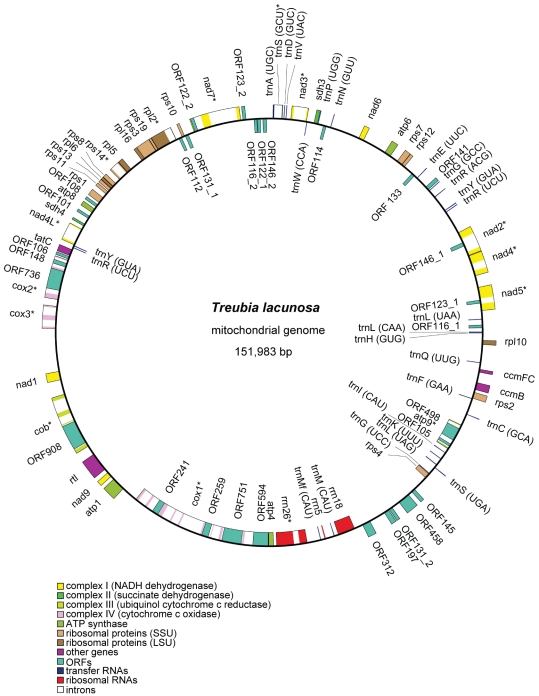
The gene map of *Treubia lacunosa* mitochondrial genome. Genes (exons indicated as closed boxes) shown on the outside of the circle are transcribed clockwise, whereas those on the inside are transcribed counter-clockwise. Genes with group I or II introns (open boxes) are labeled with asterisks. Pseudogenes are indicated with the prefix “ψ”.

**Figure 2:**
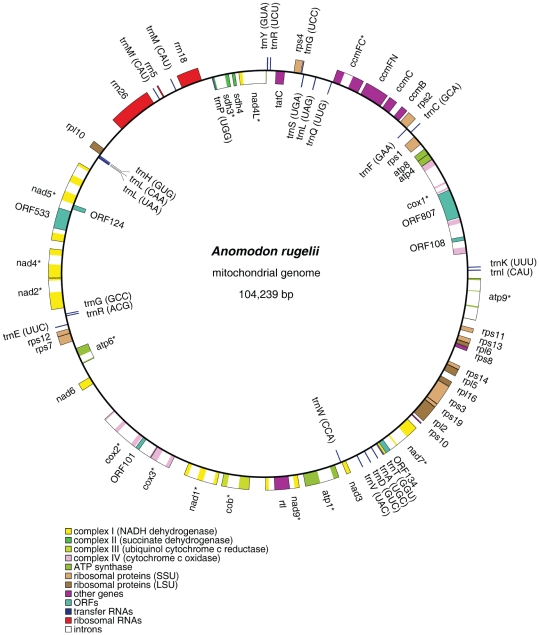
The gene map of *Anomodon rugelii* mitochondrial genome. Genes (exons indicated as closed boxes) shown on the outside of the circle are transcribed clockwise, whereas those on the inside are transcribed counter-clockwise. Genes with group I or II introns (open boxes) are labeled with asterisks. Pseudogenes are indicated with the prefix “ψ”.

Both *Treubia* and *Anomodon* chondromes are assembled as single circular molecules (Figs. 1 and 2, deposited in GenBank under accessions JF973315 and JF973314). Their sizes are 151,983 and 104,239 base pairs (bp) respectively, with AT contents of 56.6% and 58.8%. The percentages of the various sequence elements (genes, exons, introns, and intergenic spacers) in the genome are shown in Table 1.

**Table 1:** Genome sizes and proportions of the various types of sequence in the mitochondrial genomes of *Chara vulgaris* and seven bryophytes.

Species	Genome size (bp)	AT (%)	Genes (%)	Exons (%)	Introns (%)	Intergenic spacers (%)
*Chara vulgaris*	67,737	59.1	91	52	39	9
*Treubia lacunosa*	151,983	56.6	53	26	27	47
*Marchantia polymorpha*	186,609	57.6	51	23	28	49
*Pleurozia purpurea*	168,526	54.6	52	29	23	48
*Physcomitrella patens*	105,340	59.4	65	37	28	35
*Anomodon rugelii*	104,239	58.8	70	39	31	30
*Phaeoceros laevis*	209,482	55.4	47	11	36	53
*Megaceros aenigmaticus*	*184,908*	54.0	50	16	34	50

Because this is the first time that mitochondrial genome sequences from more than one species in each of the three bryophyte clades are available, and similar data have also become available from related charophytes and lycophytes recently, we will provide some comparative analyses across major lineages of early land plants on all aspects of the genomes discussed.

Comparison of the seven bryophyte chondromes with the *Chara* chondrome shows that the genome size increased roughly by three times during the origin of land plants and has been relatively stable afterwards, especially within liverworts, mosses, and hornworts (Table 1). The genome size variation seems to be more correlated with the change in proportion of intergenic spacers. The AT content in mitochondrial genomes does not show much variation in *Chara* and the bryophytes.

RNA editing seems to occur in both *Treubia* and *Anomodon* chondromes but at very low levels, as use of the standard genetic code allowed annotation of almost all protein-coding genes but a few that required reconstitution of start or stop codons and removal of internal stop codons (Table 2). The low level of editing in the *Treubia* chondrome is interesting, as its close relative *Haplomitrium* likely has a significantly higher level of editing, at least in *nad1* and *nad7* [43], [44]. Given that both taxa are relic members of an ancient lineage, such unequal levels of editing in the two taxa are difficult to explain by any known mechanisms, adding another example to the previously observed phenomenon of highly lineage-specific occurrence of RNA editing in land plant organellar genomes [45]. The low level of editing in the *Anomodon* chondrome parallels the situation in the *Physcomitrella* chondrome, where only 11 editing events occur in the entire genome [46]. However, some mosses such as *Takakia* may have high levels of editing according to a comparative analysis of *nad1* sequences [43].

**Table 2:** Start and stop codons altered by putative RNA editing in coding sequences of *Treubia lacunosa* and *Anomodon rugelii* mitochondrial genomes^1^.

Species	Gene	Start codon created	No. of stop codons removed	Stop codon created
*Treubia lacunosa*	*sdh3*		1 UAA -> CAA	
*Anomodon rugelii*	*atp1*	ACG -> AUG		CAA -> UAA
	*cox3*			CAA -> UAA
	*ccmC*			CAA -> UAA
	*ccmFN*			CAA -> UAA
	*tatC*	ACG -> AUG		

1 GTG is the start codon for *rpl16* in the *Treubia* chondrome, and is also the start codon for *nad9* and *rpl16* in the *Anomodon* chondrome.

No foreign DNA was detected in either *Treubia* or *Anomodon* mitochondrial genome. This result is consistent with those of several previous studies, which found no DNA of chloroplast or nuclear origin in any of the five bryophyte chondromes [12], [13], [14], [15], [17].

### Gene Contents

The *Treubia* and *Anomodon* mitochondrial genomes contain standard sets of protein-coding genes involved in respiration and protein synthesis as found in the chondromes of the liverworts *Marchantia* and *Pleurozia* and the moss *Physcomitrella* (Table S1 in File S1). The ribosomal and transfer RNA genes in these two chondromes are also similar to those in the liverwort and moss mitochondrial genomes sequenced before. In fact, gene content of the *Anomodon* chondrome is identical to that of the *Physcomitrella* chondrome, even for pseudogenes.

**Figure 3:**
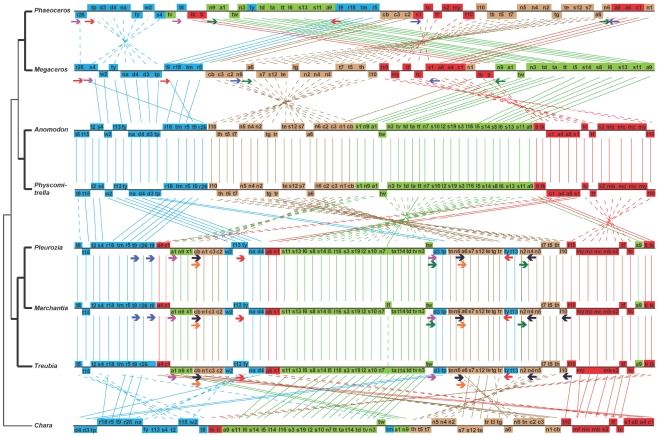
Gene order comparison among mitochondrial genomes of *Chara vulgaris, Treubia lacunosa, Marchantia polymorpha, Pleurozia purpurea, Physcomitrella patens, Anomodon rugelii, Phaeoceros laevis, and Megaceros aenigmaticus.* Species are arranged according to the organismal phylogeny [39], [40], [42], [81] except that positions of the two hornworts are reversed as the Megaceros gene order more likely represents the ancestral condition according to a parsimony criterion (a supplementary figure (Fig. S1) is presented in which the two hornworts are placed in their correct organismal phylogeny positions). Solid lines connect orthologous genes between species with the same orientation, whereas dashed lines connect those with the reversed orientation. Repeat sequences are color-coded: in liverworts, RepA – black, RepB – green, RepB2 – purple, RepC – red, RepD – blue, and RepE – orange; in hornworts, RepA – red, RepB – blue (responsible for the inversion between Megaceros and Phaeoceros), RepC – green (this class of repeats was not annotated in either hornwort due to their length of <100 bp, inverted in Megaceros but direct in Phaeoceros), and RepD – purple.

There are several aspects that deserve special attention. First, in the *Treubia* chondrome the genes involved in cytochrome *c* biogenesis are either pseudogenized (*ccmB* and *ccmFC*) or lost (*ccmC* and *ccmFN*). This is the first liverwort that has no functional mitochondrial gene encoding for this enzyme complex. Previously, two hornworts, *Megaceros* and *Phaeoceros*, were known to have no functional mitochondrial gene for cytochrome *c* biogenesis [15], [17] (Table S1 in File S1). Second, *nad7* seems to be a functional gene in *Treubia*, similar to the situation in *Haplomitrium* [44]. In all other liverworts that have been investigated, this gene is a pseudogene [44], with a functional copy residing in the nuclear genome [47]. Third, *trnTggu* is absent in *Treubia*, as in *Apotreubia*, the other genus of Treubiaceae, which was reported previously in a survey of *trnA-trnT-nad7* gene cluster in a wide variety of liverworts and mosses [48]. This gene is present on the same strand as *trnA* and *nad7* in *Chara, Blasia* (the sister group of complex thalloid liverworts to which *Marchantia* belongs), 10 diverse mosses (including *Physcomitrella* and *Anomodon*) [48], and two hornworts (in which *nad7* has been lost) [15], [17] (Fig. 3). However, in *Marchantia* and four other complex thalloid liverworts this gene is located on the opposite strand in comparison to *trnA* and *nad7*; further, it is absent in 10 other complex thalloid liverworts apparently due to a loss in their common ancestor [48]. Fourth, *rpl10*, previously known as an open reading frame (ORF) in all five sequenced bryophyte chondromes [12], [13], [14], [15], [17] and recently characterized as a functional gene encoding ribosomal protein 10 in the large subunit [49], [50], is present in both *Treubia* and *Anomodon*. Fifth, *rtl*, which codes for a reverse transcriptase, is a functional mitochondrial gene in *Treubia* as in *Marchantia*. In *Pleurozia*, *Physcomitrella*, and *Anomodon*, however, it is a pseudogene. In the two mosses and *Chara*, *rtl* is located inside a group II intron, *nad9i283* and *nad3i211* respectively, whereas in the three liverworts it is a free-standing gene positioned between *cob* and *nad9,* likely originating from a group II intron-derived reverse transcriptase gene. Finally, *trnRucu* and *trnYgua* both have a duplicated copy in the *Treubia* chondrome. The two genes are next to each other (Figs. 1 and 3) and the duplication involved both genes, which likely occurred in the ancestor of all liverworts as the same gene arrangement is found in all three sequenced liverwort chondromes. What is intriguing is that in *Marchantia*, one copy of *trnRucu* appears to have given rise to *trnRucg*, a tRNA gene that was lost in the mitochondrial genome of the common ancestor of all green plants (Chlorophyta *sensu stricto* and Streptophyta), but has been re-created from a duplicated copy of either *trnRucu* in *Marchantia* and *Chlorokybus* or *trnRacg* in *Nephroselmis* and *Mesostigma* by simply modifying the anticodon and a few other nucleotides. The detailed evidence on this finding was presented in a previous study by us when reporting the *Pleurozia* mitochondrial genome [13].

There are 20 ORFs longer than 100 codons located in intergenic spacers of the *Treubia* chondrome (Fig. 1), no such ORF was detected in the *Anomodon* chondrome (Fig. 2). In *Treubia*, the five relatively large ORFs, ORF145, ORF458, ORF131-2, ORF197, and ORF312, are located in the long intergenic spacer between *rrn18* and *rps4,* and may represent unidentified genes. Nevertheless, none of these ORFs has a homolog in *Marchantia* or *Pleurozia* and hence they may simply represent chance occurrences of reading frames. Lack of ORFs in the *Anomodon* chondrome is probably due to the overall genome size economy in the moss mitochondrion (Table 1).

Comparison of mitochondrial gene contents among three bryophyte lineages shows that liverworts and mosses are rather similar in this regard, with only minor difference in the tRNA gene complement (Table S1 in File S1). These two early lineages of land plants have mitochondrial gene contents that can rival their close charophytic alga relatives. On the other hand, hornworts have lost or are in the process of losing many genes for both respiration and protein synthesis, approaching the condition of two lycophytes sequenced so far, *Isoetes* and *Selaginella* (Table S1 in File S1).

Two recent studies have reported pseudogene pieces in intergenic spacers [48], [51]. Systematic surveys of these gene pieces are carried out here for all genes in all spacers of the seven bryophyte chondromes. The three liverwort chondromes harbor a large number of gene pieces in 30 spacers (Table S2 in File S1). The two hornwort chondromes contain only *nad6* pseudogene pieces in the *nad4-nad5* and *rrn18-trnMfcau* spacers (Table S2 in File S1). No such gene piece was found in any spacer in the two moss chondromes, almost certainly caused by the overall genome compactness in the moss mitochondria. These gene pieces are likely the results of retroposition as they lack any intron in cases of intron-containing genes (at least one intron, *cobi783*, has been retroposed separately from exons into three spacers (see below)). Most of these pieces are rather short, accounting for only a small portion of the gene. The only exceptions are the pieces for *cob* in liverworts and *nad6 in* hornworts, which account for over 80% of the gene length in both cases. In addition, some of these pieces were probably retroposed into the spacers in the common ancestor of all liverworts as they are present in the three species, and others were resulted from more recent retroposition events in more restricted scopes of taxa. Finally, some genes or portions of a gene seem to be favored targets of retroposition, as their pieces appear in more than one spacer, e.g., *atp8, ccmFC, cob, cox2, nad2*, *rpl2*, *rps7,* and *rtl* (Table S2 in File S1). No piece of ribosomal RNA or tRNA gene was detected in any spacer. At least one group II intron, *cobi783,* was retroposed (see below), but no systematic survey was conducted for introns because sequence divergence varies significantly in different domains of an intron, which makes BLAST searches difficult.

### Gene Orders and Repeat Sequences

The gene order in the *Treubia* chondrome is identical to those in the *Marchantia* and *Pleurozia* chondromes (Fig. 3). Likewise, the *Anomodon* and *Physcomitrella* mitochondrial genomes have identical gene orders. Since the three liverworts span the entire liverwort clade [40], [42], the gene order of these three species likely represents the ancestral condition of all liverworts. The two mosses cover only the diversity of the true mosses. Several other major lineages that represent relic and ancient mosses have not been sampled, i.e., Takakiales, Sphagnales, Andreaeales, Tetraphidales, Polytrichales, Buxbaumiales, and Diphysciales [39], [40]. Hence, there may not be sufficient data to infer gene order of the ancestral moss mitochondrial genome. Nevertheless, the gene orders of the two moss chondromes are not very different from those in the liverwort and hornwort chondromes (Fig. 3), which suggests that they may not be too different from the ancestral condition of the moss mitochondrial genome.

Four classes of long repeat sequences longer than 100 bp are present in the *Treubia* chondrome (Table 3). The classes A and C, both inverted repeats, have been characterized in *Pleurozia* before [13] and are also present in *Marchantia*. Two new classes, B2 and E, both direct repeats, are found in *Treubia* and *Marchantia*; they are present in *Pleurozia* but were not characterized previously because of their short length (<100 bp). The classes B and D found in *Pleurozia* and *Marchantia* are absent in *Treubia*, and probably arose after Treubiaceae/Haplomitraceae had diverged. Five of these classes (B, B2, C, D and E) have two copies each in the genome and their locations are listed in Table 3. The class A, however, has four copies in *Treubia* and *Marchantia* and three copies in *Pleurozia*. Two of these copies are closely related, sharing a long stretch of identical sequences, and are located in a group II intron (*cobi783*) and a spacer between *nad4* and *nad5*. They are present in all three species at the same locations. The additional more divergent copies are located in the *atp6*-*nad6* and *trnQuug*-*rpl10* spacers (the latter copy has been lost in *Pleurozia*) (Fig. 3).

**Table 3:** Repeat sequences in the mitochondrial genomes of *Treubia lacunosa, Marchantia polymorpha,* and *Pleurozia purpurea* 1.

*Treubia lacunosa*				*Marchantia polymorpha*				*Pleurozia purpurea*			
Name	Location	Length (bp)	Direction	Name	Location	Length (bp)	Direction	Name	Location	Length (bp)	Direction
RepA_Tl^2^	within *cobi783*, between *nad4* & *nad5*	788	inverted	RepA_Mp	within *cobi783*, between *nad4* & *nad5*	428	inverted	RepA_Pp	within *cobi783*, between *nad4* & *nad5*	446	inverted
---	---	---	---	RepB_Mp	between *sdh3* & *trnWcca*, part *nad2*	134	direct	RepB_Pp	between *sdh3* & *trnWcca*, part *nad2*	187	direct
RepB2_Tl	between *sdh3* & *trnWcca*, part *atp1*	208	direct	RepB2_Mp	between *sdh3* & *trnWcca*, part *atp1*	169	direct	RepB2_Pp	between *sdh3* & *trnWcca*, part *atp1*	65	direct
RepC_Tl^3^	between *nad4L* & *tatC*, between *nad2* & *trnRacg*	486	inverted	RepC_Mp	between *nad4L* & *tatC*, between *nad2* & *trnRacg*	154	inverted	RepC_Pp	between *nad4L* & *tatC*, between *nad2* & *trnRacg*	630	inverted
---	---	---	---	RepD_Mp	*trnMfcau* region, *trnMfcau* region	191	direct	RepD_Pp	*trnMfcau* region, *trnMfcau* region	270	direct
RepE_Tl	between *atp6* & *nad6*, part *cob*	239	direct	RepE_Mp	between *atp6* & *nad6*, part *cob*	140	direct	RepE_Pp	between *atp6* & *nad6*, part *cob*	73	direct

1 Because Genbank accepts only identical sequences as the definition of repeat sequences, the repeat length is only for the longest piece in the repeat region that has identical sequence with another piece elsewhere in the genome. There may be sequences up- and/or downstream of the piece that are parts of the repeat, which are not included for length calculation here because of nucleotide variation. Some repeats <100 bp are included here because their homologs (orthologs) in other species exceed 100 bp. All repeats have only two copies in the genome excep RepA, which has one or two additional more divergent copies in *Treubia* and *Marchantia* respectively (between *atp6* & *nad6* (*Marchantia* only), and between *trnQuug* & *trnHgug*).

2 Additional more divergent copies of RepA_Tl are located in the spacers between *atp6* & *nad6* and between *trnQuug* & *rpl10*.

3 RepC includes *trnRucu* and *trnYgua* in all three species.

One intriguing aspect about these long repeats is that all six classes involve duplication of genes or introns in intergenic spacers, probably via retroposition. Recently, it has been reported in the hornwort *Megaceros* chondrome that all three classes of long repeats involve duplication of genes or an intron [15]. Out of ten classes of repeats now known in the liverwort and hornwort chondromes, only one class (D in *Phaeoceros*) does not involve duplication of any gene or intron [17]. There is no homology between the repeat sequences in the liverworts and the hornworts.

No long repeat sequence was found in the *Anomodon* chondrome. Short (<100 bp) repeat sequences were identified in both *Treubia* and *Anomodon* chondromes. In the former, they are of diverse sequence compositions, whereas in the latter they are mostly domains V and VI or other elements of closely related group II introns (data not shown). Only one family of microsatellite sequence, AT dinucleotides reiterating 10 times in the second exon of *cob*, was found in the *Anomodon* chondrome. No such sequence was detected in the *Treubia* chondrome.

Though there are no or only a few changes in mitochondrial gene order within each of the three bryophyte lineages as shown by the seven sequenced chondromes, a minimum of 17 and 6 changes (inversions and translocations) need to be invoked to explain the gene order differences among liverworts, mosses, and hornworts (Fig. 3). How these genomic rearrangements happened are difficult historical events to reconstruct. Repeat sequences have been suggested to serve as sites of homologous recombination, resulting gene order changes in organellar genomes [52], [53], [54]. A pair of long repeat sequences were recently identified in the *Megaceros* and *Phaeoceros* chondromes that were located outside a region that had been inverted [17]. The repeat sequences detected in the liverwort chondromes in this and an earlier study [13] may have been responsible for mitochondrial gene order changes between liverworts and other early land plants. One class, repeat A, is particularly noteworthy (Table 3). A major block of genes, including many encoding ribosomal proteins, respiratory proteins, and some tRNAs, are located on opposite strands between *Chara*-liverworts and mosses-hornworts (Fig. 3). The former condition can be traced back to *Cyanidioschyzon merolae* (a unicellular red alga) [55] and *Nephroselmis olivacea* (a unicellular prasinophyceae green alga) [56] (also see Fig. 2 in [15]), whereas the latter condition extends to *Huperzia squarrosa* (a basal lycophyte and an early vascular plant; Y. Liu, B. Wang, P. Cui, L. Li, J.-Y. Xue, J. Yu, & Y.-L. Qiu, unpublished data). It is thus possible that the *Chara*-liverworts gene arrangement represents the ancestral condition and that of the mosses and hornworts is an evolutionarily derived condition. This change was perhaps caused by a major inversion within the mitochondrial genome of the common ancestor of mosses-hornworts-vascular plants after liverworts had diverged. The class A repeats, likely present in the chondrome of that common ancestor and retained in the modern liverwort chondromes, probably served as sites of homologous recombination. The positions of the two members, one located in the second intron in *cob* and the other in the spacer between *trnQuug* and *rpl10*, match roughly but rather nicely with the boundary points defining the region that has undergone inversion (Fig. 3). Two facts are consistent with this hypothesis. One is that the *trnQuug*-*rpl10* copy shows a higher level of divergence to the *cob* intron copy than the *nad4-nad5* spacer copy, indicating its more ancient history. Additionally, most members of this class are present in the three diverse liverworts, again indicating their ancient history. One might argue that absence of this class of repeats in the moss and hornwort chondromes does not support the hypothesis. However, this evidence can be used to explain evolutionary fixation of rearranged gene orders in the chondromes of mosses, hornworts, and basal vascular plants, which indeed have not reverted back to the *Chara*-liverwort condition. Further, the moss chondromes are rather economical in size (Table 1), and repeat sequences might have been purged from the genome shortly after the origin of mosses. It would be desirable to sequence chondromes of some basal moss lineages such as *Takakia* and *Polytrichum* to see if they have larger genomes and harbor long repeat sequences.

Evolution of gene order in bryophyte chondromes is overall quite conservative, both within and among major lineages. The extent of conservation is especially striking when compared with rapidly rearranged mitochondrial genomes of vascular plants [18], [19], [21], [23], [25], [26], [28]. A total of 19, 18 and 7 inversions and translocations (plus some deletions) can explain gene order differences of chondromes between *Chara* and *Treubia, Pleurozia* and *Physcomitrella,* and *Anomodon* and *Megaceros* respectively (Fig. 3). These are very modest number of changes for the lineages that have existed for at least 375 million years, the age of liverworts inferred from a well-preserved fossil [57]. In sharp contrast, two cytotypes of maize differ by 16 rearrangements in their chondromes [23]. If any adaptive explanation is sought, which has been explored in several recent studies of molecular evolution in organellar genomes [45], [58], [59], one wonders what are the reasons behind such high levels of conservation. One factor may be that organellar genomes, like their ancestral bacterial genomes, have polycistronic operons [60], [61], [62], [63], [64]. Some gene clusters, which likely represent polycistronic operons, have widespread phylogenetic distribution in green algae and early land plants, with some even found in *Reclinomonas americana* (a basal eukaryote) [65] or *Cyanidioschyzon merolae* [55]. These include rRNA genes, ribosomal protein genes, *nad2-nad4-nad5*, *ccm* genes, *nad4L-sdh* genes, and *cox2-cox3-cob-nad6* [15]. Expression of these genes has probably exerted strong functional selection pressure on tight linkage and proper arrangement order of these genes in certain regions of the mitochondrial genome. Further, the products of these genes are assembled into protein/enzyme complexes involved in respiration and protein synthesis together with those encoded by nuclear genes, which were originally mitochondrial genes that migrated to the nucleus during post-endosymbiosis evolution [66]. This process would add another layer of functional constraint on inheritance and expression of the organellar genes. Recently, some nuclear genes encoding proteins that suppress recombination in mitochondria and chloroplasts in the plant cell have been characterized in *Physcomitrella* and some angiosperms [54], [67], [68]. Thus, conservative gene order evolution in organellar genomes seems to be resulted from both historical/genome structural reasons and functional non-autonomy of these tiny genomes. Nevertheless, these reasons are insufficient to explain the extreme level of conservation in the bryophyte mitochondrial genomes, because the same organellar genome is radically recombinogenic in vascular plants. Hence, additional, likely organismal, explanations need to be sought to account for such drastically different levels of gene order conservation in bryophyte and vascular plant chondromes.

### Intron Contents

The intron content of the *Treubia* chondrome is similar to those in the *Marchantia* and *Pleurozia* chondromes. Both group I and group II introns are present and they are all *cis*-spliced (Table S3 in File S1). However, *Treubia* does not have either of the two group II introns (*atp1i989* and *atp1i1050*) found in *atp1* of *Marchantia* and *Pleurozia*. Likewise, it lacks one of the two group II introns in *nad4L* (*nad4Li283*) of the other two liverworts. The sole group II intron in *Marchantia rrn18* (*rrn18i1065*) is also lacking in *Treubia*, as in *Pleurozia*.

The intron content in the *Anomodon* chondrome is exactly the same as that in the *Physcomitrella* chondrome, again all being *cis*-spliced, whether they are group I or group II (Table S3 in File S1).

With intron contents determined from the seven completely sequenced bryophyte chondromes, it is clear that the largely unique intron content in each of the three bryophyte lineages is shared by most members of the lineage and can be attributed to independent gains of these mobile genetic elements in the common ancestors of liverworts, mosses, and hornworts respectively (Table S3 in File S1). Secondary losses and later acquisitions seem to have happened, but only sporadically. This pattern of intron distribution is consistent with the rather conservative mode of gene order and gene content evolution in liverworts, mosses, and hornworts.

### Dynamic and Conservative Evolution of Mitochondrial Genomes in Early Land Plants

The data from the *Treubia* and *Anomodon* chondromes reinforce the conclusion of several recent studies that mitochondrial genomes in early land plants show a mixed mode of dynamic and conservative evolution [13], [14], [15], [17]. During the origin of land plants, changes in the following aspects were quite dynamic: genome size increase, gene order change, gene structural alteration (*ccmF* fractured into *ccmFC* and *ccmFN*), and appearance of RNA editing machinery. The dynamic mode of evolution continued as major clades of land plants appeared: genome size increase or decrease, massive waves of intron acquisition, extensive gene losses in hornworts, generation of repeat sequences, and major inversions and translocations resulting gene order change. On the other hand, several aspects of the genome were rather conservative during this phase of plant evolution, in particular in comparison with vascular plant chondromes. These include: overall genome size, genome structure (lack of recombination that generated subgenomic circles), sequence composition (AT%), gene order, and genome content (lack of foreign DNA). Within liverworts, mosses, and hornworts, conservative evolution of mitochondrial genomes was even more extreme. Not only gene and intron contents varied little among diverse species within each of the three major clades of bryophytes, pseudogene gene contents and retroposed pseudogene pieces were highly similar as well. Gene orders were also identical among different species of liverworts and mosses respectively, and those of two hornworts were also rather similar. Before these bryophyte chondromes were sequenced, no prediction could be made about what they might look like based on what was known of mitochondrial genomes in angiosperms and pteridophytes [25], [28], [31], [33], [69].

With at least two species sequenced for their chondromes in each of the three bryophyte lineages, most features of this organellar genome seem to follow some patterns within the lineage. However, RNA editing, which is correlated to changes in protein hydrophobicity and molecular size in land plant organellar genomes [45], remains unpredictable, as its abundance can vary dramatically in closely related taxa such as *Treubia* and *Haplomitrium*. In lycophytes, similarly highly disparate occurrence of RNA editing has been observed in *Isoetes* [38] and *Selaginella* [19] versus *Huperzia* (Y. Liu, B. Wang, P. Cui, L. Li, J.-Y. Xue, J. Yu, & Y.-L. Qiu, unpublished data). Underlying causes of this enigmatic molecular evolutionary phenomenon need to be pursued in future studies.

Finally, what may be speculated about is that an evolutionarily derived type of mitochondrial genome expression system seems to have evolved in vascular plants, as bryophytes still have the typical and ancestral type of mitochondrial genomes. Notably, the genes in bryophyte chondromes remain organized into long polycistronic operons as in green and red algae as well as the basal eukaryote *Reclinomonas* [8], [10], [11], [56], [65], despite many changes that took place during the origin of land plants and emergence of liverworts, mosses, and hornworts. Frequent intragenomic rearrangements in vascular plant chondromes have broken most of these operons and many genes become free-standing as in the nuclear genome, which may be caused by mutations of nuclear genes identified recently that suppress recombination in organellar genomes [54], [67], [68]. During the bryophyte-vascular plant transition, the dominant generation in the plant life cycle changed from a haploid gametophyte to a diploid sporophyte, plant size increased by several orders of magnitude, and the physical environment changed correspondingly [35], [70], [71], [72], [73]. The change revealed in mitochondria, the powerhouse of the plant cell, is correlated to this major change in evolution of land plants and the environment on earth; whether there is any causative relationship between them is currently unknown.

## Materials and Methods

Approximately 10 g of fresh tissues of *Treubia lacunosa* (Col.) Prosk. and *Anomodon rugelii* (C.M.) Keissl. were collected in the field from New Zealand and Michigan, USA, respectively. The *Treubia* material was collected with a permit (Number BP-17540-FLO) to Dr. Matt von Konrat at Field Museum (USA) issued and coordinated by Paul Cashmore of the New Zealand Department of Conservation. The *Anomodon* material was collected on public land, requiring no permit because it is a common weedy species. The material was brought to the lab for cleaning under a dissecting scope. For *Treubia*, a voucher specimen numbered John J. Engel & Matt von Konrat 28345 was deposited at Field Museum. For *Anomodon*, a voucher specimen numbered Qiu 06002 was deposited at the University Herbarium in the University of Michigan, Ann Arbor.

Total cellular DNA was extracted with the CTAB method [74], and purified with phenol extraction to remove proteins. A fosmid library was constructed using the CopyControl^TM^ kit (EPICENTRE Biotechnologies, Madison, Wisconsin, USA) from the total cellular DNA fragments of 35–45 kb size-selected by agarose gel electrophoresis. No restriction enzyme digestion or mechanical shearing was used before electrophoresis. Clones containing mitochondrial DNA fragments were identified through Southern hybridizations using the HRP chemiluminescent blotting kit (KPL, Inc., Gaithersburg, Maryland, USA), with major mitochondrial genes as probes. The probes were made by amplification from total cellular DNA of *Treubia* or *Anomodon.* The inserts were sequenced through primer-walking on an ABI 3100 genetic analyzer (Applied Biosystems, Foster City, California, USA). Sequences were assembled using Sequencher (Gene Codes Corp., Ann Arbor, Michigan, USA).

The mitochondrial genomes were annotated in six steps. First, genes for known mitochondrial proteins and rRNAs were identified by Basic Local Alignment Search Tool (BLAST) searches [75] (http://www.ncbi.nlm.nih.gov/blast/Blast.cgi) of the non-redundant database at the National Center for Biotechnology Information (NCBI). The exact gene and exon/intron boundaries were predicted by alignment of orthologous genes from annotated plant mitochondrial genomes available at the organelle genomic biology website at NCBI (http://www.ncbi.nlm.nih.gov/genomes/ORGANELLES/organelles.html). Putative RNA editing sites were inferred to create proper start and stop codons as well as to remove internal stop codons. Second, genes for hypothetical proteins were identified using the web-based tool Open Reading Frames Finder (ORF-finder; http://www.ncbi.nlm.nih.gov/gorf/gorf.html) with the standard genetic code. Third, genes for tRNAs were found using tRNAscan-SE [76] (http://lowelab.ucsc.edu/tRNAscan-SE/). Fourth, repeated sequences were searched using REPuter [77] (http://bibiserv.techfak.uni-bielefeld.de/reputer/) or BLAST. Fifth, microsatellite sequences were screened using msatcommander 0.8.2 with default settings [78]. Finally, pseudogene pieces in intergenic spacers were identified by BLASTing gene sequences against spacer sequences, and those longer than 50 bp were recorded in this study.

The annotated GenBank files of the mitochondrial genomes of *Treubia* and *Anomodon* were used to draw gene maps by using OrganellarGenomeDRAW tool (OGDRAW) [79]. The maps were then examined for further comparison of gene order and content. When sequence homology in some parts of certain genes or intergenic spacers was uncertain, the sequences were aligned using CLUSTAL_X [80], with visual examination followed.

## Supporting Information

Figure S1 Gene order comparison among mitochondrial genomes of *Chara vulgaris, Treubia lacunosa*, *Marchantia polymorpha, Pleurozia purpurea, Physcomitrella patens, Anomodon rugelii, Phaeoceros laevis,* and *Megaceros aenigmaticus*. Species are arranged according to the organismal phylogeny [39], [40], [42], [81]. Solid lines connect orthologous genes between species with the same orientation, whereas dashed lines connect those with the reversed orientation. Repeat sequences are color-coded: in liverworts, RepA – black, RepB – green, RepB2 – purple, RepC – red, RepD – blue, and RepE – orange; in hornworts, RepA – red, RepB – blue (responsible for the inversion between *Megaceros* and *Phaeoceros*), RepC – green (this class of repeats was not annotated in either hornwort due to their length of <100 bp, inverted in *Megaceros* but direct in *Phaeoceros*), and RepD – purple.
(EPS)

File S1 Contains **Table S1.** Gene contents in mitochondrial genomes of selected charophyte and land plants^1^. **Table S2.** Pseudogene pieces in intergenic spacers of mitochondrial genomes of *Treubia lacunosa, Marchantia polymorpha, Pleurozia purpurea*, *Phaeoceros laevis, and Megaceros aenigmaticus* ^1^. **Table S3.** Intron contents in mitochondrial genomes of selected charophyte and land plants.^1^
(DOCX)
