# Supplementary material for: The Mitochondrial Genomes of the Early Land Plants Treubia lacunosa and Anomodon rugelii: Dynamic and Conservative Evolution
Source: PLoS One. 2011 Oct 5;6(10):e25836. doi: 10.1371/journal.pone.0025836 (PMC3187804; doi:10.1371/journal.pone.0025836)
Supplement: File S1 — Contains Table S1. Gene contents in mitochondrial genomes of selected charophyte and land plants1. Table S2. Pseudogene pieces in intergenic spacers of mitochondrial genomes of Treubia lacunosa, Marchantia polymorpha, Pleurozia purpurea, Phaeoceros laevis, and Megaceros aenigmaticus 1. Table S3. Intron contents in mitochondrial genomes of selected charophyte and land plants.1 (DOCX) [file pone.0025836.s002.docx]

**Table S1.** Gene contents in mitochondrial genomes of selected charophyte and land plants^1^.

| **gene/species** |  | ***Ch. vu.*** | ***Tr. la.*** | ***Ma. po.*** | ***Pl. pu.*** | ***Ph. pa.*** | ***An. ru.*** | ***Ph. la.*** | ***Me. ae.*** | ***Is. en.*** | ***Se*. *mo*.** |
| --- | --- | --- | --- | --- | --- | --- | --- | --- | --- | --- | --- |
| *atp1* | a1 | + | + | + | + | + | + | + | + | + | + |
| *atp4* | a4 | + | + | + | + | + | + | + | + | + |  |
| *atp6* | a6 | + | + | + | + | + | + | + | + | + | + |
| *atp8* | a8 | + | + | + | + | + | + | ψ | ψ | + | + |
| *atp9* | a9 | + | + | + | + | + | + | + | + | + | + |
| *ccmB* | mb | + | ψ | + | + | + | + |  |  |  |  |
| *ccmC* | mc | + |  | + | + | + | + |  |  |  |  |
| *ccmF*^2^ | mf | + |  |  |  |  |  |  |  |  |  |
| *ccmFC*^2^ | my |  | ψ | + | + | + | + | ψ | ψ |  |  |
| *ccmFN*^2^ | mz |  |  | + | + | + | + |  |  |  |  |
| *cob* | cb | + | + | + | + | + | + | + | + | + | + |
| *cox1* | c1 | + | + | + | + | + | + | + | + | + | + |
| *cox2* | c2 | + | + | + | + | + | + | + | + | + | + |
| *cox3* | c3 | + | + | + | + | + | + | + | + | + | + |
| *nad1* | n1 | + | + | + | + | + | + | + | + | + | + |
| *nad2* | n2 | + | + | + | + | + | + | + | + | + | + |
| *nad3* | n3 | + | + | + | + | + | + | + | + | + | + |
| *nad4* | n4 | + | + | + | + | + | + | + | + | + | + |
| *nad4L* | na | + | + | + | + | + | + | + | + | + | + |
| *nad5* | n5 | + | + | + | + | + | + | + | + | + | + |
| *nad6* | n6 | + | + | + | + | + | + | + ψ | + ψ | + | + |
| *nad7* | n7 | + | + | ψ | ψ | + | + |  |  | + | + |
| *nad9* | n9 | + | + | + | + | + | + | + | + | + | + |
| *rpl2* | l2 | + | + | + | + | + | + |  |  |  |  |
| *rpl5* | l5 | + | + | + | + | + | + |  | ψ | + |  |
| *rpl6* | l6 | + | + | + | + | + | + | ψ | ψ |  |  |
| *rpl10* | l10 |  | + | + | + | + | + | + | + |  |  |
| *rpl14* | l14 | + |  |  |  |  |  |  |  |  |  |
| *rpl16* | l16 | + | + | + | + | + | + |  |  |  |  |
| *rps1* | s1 | + | + | + | + | + | + | ψ | ψ | ψ |  |
| *rps2* | s2 | + | + | + | + | + | + | ψ |  | + |  |
| *rps3* | s3 | + | + | + | + | + | + |  |  | + |  |
| *rps4* | s4 | + | + | + | + | + | + | ψ | ψ | + |  |
| *rps7* | s7 | + | + | + | + | + | + | ψ | ψ |  |  |
| *rps8* | s8 |  | + | + | + | ψ | ψ |  | ψ |  |  |
| *rps10* | s10 | + | + | + | + | ψ | ψ |  |  |  |  |
| *rps11* | s11 | + | + | + | + | + | + | ψ | ψ |  |  |
| *rps12* | s12 | + | + | + | + | + | + | ψ | ψ |  |  |
| *rps13* | s13 |  | + | + | + | + | + | + | + |  |  |
| *rps14* | s14 | + | + | + | + | + | + |  | + |  |  |
| *rps19* | s19 | + | + | + | + | + | + |  |  |  |  |
| *rrn5* | r5 | + | + | + | + | + | + | + | + | + |  |
| *rrn18* | r18 | + | + | + | + | + | + | + | + | + | + |
| *rrn26* | r26 | + | + | + | + | + | + | + | + | + | + |
| *rtl*^3^ | x1 | + | + | + | ψ | ψ | ψ |  |  |  |  |
| *sdh3* | d3 | + | + | + | + | + | + | ψ | ψ | + |  |
| *sdh4* | d4 | + | + | + | + | + | + | + | + |  |  |
| *tatC* | w2 | + | + | + | + | + | + | + | + | + | + |
| *trnAugc* | ta | + | + | + | + | + | + | + | + |  |  |
| *trnCgca* | tc | + | + | + | + | + | + | + | + | + |  |
| *trnDguc* | td | + | + | + | + | + | + | + | + |  |  |
| *trnEuuc* | te | + | + | + | + | + | + | + | + | + |  |
| *trnFgaa* | tf | + | + | + | + | + | + | + | + | + |  |
| *trnGgcc* | tg | + | + | + | + | + | + | + | + | + |  |
| *trnGucc* | t2 | + | + | + | + | + | + |  |  |  |  |
| *trnHgug* | th | + | + | + | + | + | + | + | + |  |  |
| *trnIcau* | ti | + | + | + | + | + | + | + | + | + |  |
| *trnIgau* | t3 | + |  |  |  |  |  |  |  |  |  |
| *trnKuuu* | tk | + | + | + | + | + | + | + | + | + |  |
| *trnLcaa* | t5 | + | + | + | + | + | + | + | + |  |  |
| *trnLuaa* | t7 | + | + | + | + | + | + | + | + |  |  |
| *trnLuag* | t8 | + | + | + | + | + | + | + |  | ψ |  |
| *trnMcau* | tm | + | + | + | + | + | + | + | + | + |  |
| *trnMfcau* | t9 | + | + | ++ | ++ | + | + | + | + | + |  |
| *trnNguu* | tn | + | + | + | + |  |  |  |  |  |  |
| *trnPugg* | tp | + | + | + | + | + | + | + | + | + |  |
| *trnQuug* | t10 | + | + | + | + | + | + | + | + | + |  |
| *trnRacg* | tr | + | + | + | + | + | + |  |  |  |  |
| *trnRucg* | t12 |  |  | + |  |  |  |  |  |  |  |
| *trnRucu* | t13 | + | ++ | + | ++ | + | + |  |  |  |  |
| *trnSgcu* | t14 | + | + | + | + |  |  |  |  |  |  |
| *trnSuga* | t15 | + | + | + | + | + | + |  |  | + |  |
| *trnTggu* | tt | + |  | + |  | + | + | + | + |  |  |
| *trnVuac* | tv | + | + | + | + | + | + | + |  |  |  |
| *trnWcca* | tw | + | + | + | + | + | + | + | + | + |  |
| *trnYgua* | ty | + | ++ | ++ | ++ | + | + | + ψ | + | + |  |

#### ^1^The second column represents abbreviated gene names used in Figure 3. The full species names are as follows (in the order as they appear): *Chara vulgaris, Treubia lacunosa*, *Marchantia polymorpha, Pleurozia purpurea, Physcomitrella patens, Anomodon rugelii, Phaeoceros laevis, Megaceros aenigmaticus, Isoetes engelmannii,* and *Selaginella moellendorffii*. “+” or “ψ” indicate presence of a functional gene or a pseudogene respectively. Two plus signs indicates presence of a duplicated copy.

^2^The genes *ccmFC* and *ccmFN* of land plants appear as a single gene *ccmF* in *Chara*.

^3^The gene *rtl* is located inside a group II intron, *nad3i211* and *nad9i283* in *Chara* and the two mosses respectively*.*

**Table S2.** Pseudogene pieces in intergenic spacers of mitochondrial genomes of *Treubia lacunosa, Marchantia polymorpha, Pleurozia purpurea*, *Phaeoceros laevis, and Megaceros aenigmaticus*^1^*.*

| **Spacer** | ***Treubia lacunosa*** | | | | | ***Marchantia polymorpha*** | | | | | ***Pleurozia purpurea*** | | |
| --- | --- | --- | --- | --- | --- | --- | --- | --- | --- | --- | --- | --- | --- |
|  | **Gene piece^2^** | **Length (bp)** | | **Identity^3^** | | **Gene piece^2^** | **Length (bp)** | | **Identity^3^** | | **Gene piece^2^** | **Length (bp)** | **Identity^3^** |
| *atp1-cox1* |  |  | |  | | *ccmFC* (383-441) | 59 | | 92% | |  |  |  |
| *atp4-trnMf* |  |  | |  | |  |  | |  | | *atp8* (3-79) | 77 | 90% |
| *atp6-nad6* | *cob* (3-487) | 477 | | 96% | | *cob* (3-706) | 676 | | 92% | | *cob* (1-696) | 653 | 86% |
|  |  |  | |  | | *cob* (776-1157) | 375 | | 89% | | *cob* (784-1140) | 356 | 87% |
| *atp8-sdh4* | *atp8* (1-199) | 203 | | 75% | | *atp8* (3-181) | 185 | | 79% | | *atp8* (3-189) | 193 | 78% |
|  | *nad2* (1020-1086) | 67 | | 76% | | *nad2* (927-1086) | 126 | | 69% | | *nad2* (927-1086) | 126 | 71% |
|  | *nad2* (1375-1467) | 93 | | 85% | | *nad2* (1375-1470) | 96 | | 98% | | *nad2* (1375-1470) | 96 | 90% |
|  | *rps7* (41-284) | 250 | | 84% | | *rps7* (41-293) | 252 | | 98% | | *rps7* (41-287) | 248 | 97% |
| *ccmC-ccmFN* |  |  | |  | | *ccmFC* (318-385) | 67 | | 93% | |  |  |  |
| *ccmFN-ccmFC* |  |  | |  | | *ccmFC* (366-454) | 89 | | 75% | |  |  |  |
| *cob-rtl* |  |  | |  | | *nad9* (6-120) | 116 | | 92% | |  |  |  |
|  |  |  | |  | | *rtl* (554-630) | 77 | | 91% | |  |  |  |
|  |  |  | |  | | *rtl* (569-628) | 58 | | 76% | |  |  |  |
| *cox2-cox3* | *cox2* (671-759) | 89 | | 93% | | *cox2* (668-755) | 88 | | 91% | | *cox2* (668-754) | 87 | 94% |
| *cox3-nad1* |  |  | |  | | *ccmFC* (389-461) | 68 | | 82% | |  |  |  |
| *nad1-cob* |  |  | |  | | *ccmFC* (386-436) | 51 | | 94% | |  |  |  |
|  |  |  | |  | |  |  | |  | | *cox2* (694-755) | 62 | 81% |
|  |  |  | |  | | *cob* (1052-1138) | 88 | | 80% | | *cob* (1053-1137) | 84 | 77% |
| *nad2-trnRucu* | *tatC* (1-94) | 94 | | 99% | | *tatC* (1-106) | 106 | | 94% | | *tatC* (1-107) | 107 | 99% |
| *nad3-trnVuac* |  |  | |  | | *rtl* (558-616) | 68 | | 81% | |  |  |  |
| *nad6-trnNguu* | *atp6* (1-175) | 163 | | 87% | | *atp6* (1-183) | 177 | | 87% | | *atp6* (1-183) | 186 | 82% |
| *nad7-rps10* | *rtl* (531-585) | 56 | | 84% | | *rtl* (551-627) | 77 | | 87% | |  |  |  |
|  |  |  | |  | | *rpl2* (727-798) | 72 | | 82% | |  |  |  |
|  |  |  | |  | | *ccmFC* (335-424) | 84 | | 79% | |  |  |  |
| *nad9-atp1* |  |  | |  | | *rtl* (545-615) | 71 | | 94% | |  |  |  |
| *rpl16-rpl5* | *rtl* (1368-1426) | 68 | | 79% | |  |  | |  | |  |  |  |
|  |  |  | |  | | *ccmFC* (363-450) | 82 | | 81% | | *ccmFC* (366-428) | 63 | 83% |
| *rps7-atp6* |  |  | |  | | *ccmFC* (1133-1188) | 56 | | 93% | |  |  |  |
| *rps11-rps1* |  |  | |  | | *ccmFC* (1117-1187) | 64 | | 85% | | *ccmFC* (1102-1166) | 65 | 80% |
| *rps14-rps8* |  |  | |  | | *ccmFC* (329-434) | 113 | | 83% | |  |  |  |
| *rrn18-rps4* |  |  | |  | | *rtl* (564-624) | 62 | | 81% | |  |  |  |
| *rrn26-trnMfcau* | *atp8* (1-84) | 84 | | 87% | | *atp8* (3-84) | 82 | | 87% | | *atp8* (3-72) | 70 | 93% |
| *sdh3-trnWcca* | *atp1* (1336-1542) | 207 | | 100% | | *atp1* (1195-1542) | 348 | | 99% | | *atp1* (1195-1542) | 342 | 94% |
|  |  |  | |  | | *nad2* (1104-1470) | 366 | | 98% | | *nad2* (1104-1470) | 367 | 96% |
| *sdh4-nad4L* |  |  | |  | |  |  | |  | | *rpl2* (669-719) | 56 | 86% |
| *trnAugc-nad7* | *rps7* (105-160) | 56 | | 96% | |  |  | |  | | *rps7* (98-169) | 72 | 90% |
| *trnLuaa-trnLcaa* | *nad2* (1093-1331) | 236 | | 90% | | *nad2* (1093-1331) | 238 | | 96% | | *nad2* (1093-1331) | 239 | 92% |
| *trnMcau-rrn18* |  |  | |  | | *nad6* (1-229) | 293 | | 89% | | *nad6* (1-314) | 319 | 78% |
|  |  |  | |  | |  |  | |  | | *cox1* (1337-1558) | 207 | 80% |
| *trnNguu-sdh3* |  |  | |  | | *rtl* (559-618) | 61 | | 70% | |  |  |  |
|  |  |  | |  | | *rtl* (631-682) | 51 | | 85% | |  |  |  |
| *trnQuug-trnHgug* |  |  | |  | | *ccmFC* (382-447) | 69 | | 86% | |  |  |  |
| *trnSuga-trnLuag* |  |  | |  | | *ccmFC* (366-449) | 77 | | 80% | | *ccmFC* (366-423) | 58 | 90% |
|  |  |  | |  | |  |  | |  | | *rpl2* (669-719) | 52 | 90% |
| *trnTggu-nad7* |  |  | |  | | *nad5* (2-176) | 175 | | 87% | |  |  |  |
|  |  |  | |  | | *rps7* (98-169) | 72 | | 92% | |  |  |  |
|  |  |  | |  | | *rtl* (502-565) | 67 | | 74% | |  |  |  |
|  | ***Phaeoceros laevis*** | | | | | ***Megaceros aenigmaticus*** | | | | |  |  |  |
|  | **Gene piece^2^** | | **Length (bp)** | | **Identity^3^** | **Gene piece^2^** | | **Length (bp)** | | **Identity^3^** |  |  |  |
| *nad4-nad5* | *nad6*^4^ (1-541) | | 543 | | 76% | *nad6*^4^ (1-154) | | 512 | | 72% |  |  |  |
| *rrn18-trnMfcau* | *nad6* (17-322) | | 313 | | 78% | *nad6* (12-285) | | 286 | | 67% |  |  |  |
|  | *nad6* (452-564) | | 118 | | 76% | *nad6* (452-587) | | 148 | | 71% |  |  |  |

^1^Only gene pieces longer than 50 bp in intergenic spacers are reported here. A small number of gene pieces longer than 50 bp were detected in introns but are not reported here because *rtl*, being a group II intron-derived gene, makes such analysis difficult. The gene *rtl* is a pseudogene in *Pleurozia*, and *nad7* is a pseudogene in *Marchantia* and *Pleurozia*.

^2^The numbers following the gene name in the parenthesis indicate the range of the functional CDS to which the pseudogene piece matched.

^3^The identity is calculated by dividing the number of identical nucleotides by the aligned length of the pseudo- and functional genes.

^4^The pseudo-*nad6* within the *nad4-nad5* spacer is a joint piece of exons 1 and 2, presumably resulted from a single retroposition event.

**Table S3.** Intron contents in mitochondrial genomes of selected charophyte and land plants.^1^

| **intron/species** | ***Ch. vu.*** | ***Tr. la.*** | ***Ma. po.*** | ***Pl. pu.*** | ***Ph. pa.*** | ***An. ru.*** | ***Ph. la.*** | ***Me. ae.*** | ***Is. en.*** | ***Se. mo.*** |
| --- | --- | --- | --- | --- | --- | --- | --- | --- | --- | --- |
| *atp1i805g2* |  |  |  |  |  |  | + | + |  |  |
| *atp1i989g2* |  |  | + | + |  |  |  |  |  |  |
| *atp1i1019g2* |  |  |  |  |  |  | + | + |  |  |
| ***atp1i1050g2***** |  |  | + | + |  |  | + | + |  |  |
| *atp1i1129g2* |  |  |  |  | + | + |  |  |  |  |
| ***atp6i80g2***** |  |  |  |  | + | + | + | + |  |  |
| ***atp6i439g2***** |  |  |  |  |  |  | + | + | + | + |
| ***atp9i21g2***** |  |  |  |  | + | + |  |  | + | *trans* |
| ***atp9i87g2****** |  | + | + | + | + | + |  |  | + | + |
| ***atp9i95g2****** |  |  |  |  | + | + | + | + |  | + |
| *atp9i145g2* | + |  |  |  |  |  |  |  |  |  |
| *atp9i214g2* | + |  |  |  |  |  |  |  |  |  |
| ***ccmFCi829g2***** |  |  |  |  | + | + | + | + |  |  |
| *cobi274g2* | + |  |  |  |  |  |  |  |  |  |
| *cobi372g2* |  | + | + | + |  |  |  |  |  |  |
| ***cobi420g1***** |  |  |  |  | + | + | + |  |  |  |
| *cobi537g2* | + |  |  |  |  |  |  |  |  |  |
| *cobi688g2* | + |  |  |  |  |  |  |  |  |  |
| *cobi693g2* |  |  |  |  |  |  |  |  | + | + |
| *cobi783g2* |  | + | + | + |  |  |  |  |  |  |
| ***cobi787g2***** |  |  |  |  |  |  | + | + | + | *trans* |
| *cobi824g2* |  | + | + | + |  |  |  |  |  |  |
| *cobi838g2* |  |  |  |  |  |  | + | + |  |  |
| ***cox1i44g2***** |  | + | + | + |  |  | + | + |  |  |
| *cox1i150g2* |  |  |  |  |  |  | + | + |  |  |
| *cox1i178g2* |  | + | + | + |  |  |  |  |  |  |
| *cox1i211g2* | + |  |  |  |  |  |  |  |  |  |
| *cox1i227g2* |  |  |  |  |  |  |  |  | + | + |
| *cox1i266g2* |  |  |  |  |  |  |  |  | + | + |
| *cox1i323g2* |  |  |  |  |  |  |  |  | + |  |
| *cox1i375g1* |  | + | + | + |  |  |  |  |  |  |
| ***cox1i395g1***** |  | + | + | + |  |  |  |  | + |  |
| ***cox1i511g2****** |  | + | + | + | + | + |  |  |  | + |
| ***cox1i624g1***** |  | + | + | + | + | + |  |  |  |  |
| ***cox1i729g1***** | + | + | + | + |  |  |  |  |  |  |
| *cox1i732g2* |  |  |  |  | + | + |  |  |  |  |
| *cox1i740g1* | + |  |  |  |  |  |  |  |  |  |
| *cox1i835g2* | + |  |  |  |  |  |  |  |  |  |
| ***cox1i876g1***** | + |  |  |  |  |  |  |  |  | + |
| *cox1i909g1* | + |  |  |  |  |  |  |  |  |  |
| *cox1i995g2* |  |  |  |  |  |  |  |  | + | + |
| *cox1i1064g2* |  |  |  |  | + | + |  |  |  |  |
| *cox1i1116g1* |  | + | + | + |  |  |  |  |  |  |
| *cox1i1149g2* |  |  |  |  |  |  |  |  |  | + |
| *cox1i1298g2* |  |  |  |  |  |  | + | + |  |  |
| ***cox1i1305g1***** |  | + | + | + |  |  |  |  | *trans* | *trans* |
| *cox2i94g2* |  |  |  |  |  |  |  |  | + | + |
| *cox2i97g2* |  | + | + | + |  |  |  |  |  |  |
| ***cox2i104g2***** | + |  |  |  | + | + |  |  |  |  |
| *cox2i250g2* |  | + | + | + |  |  |  |  |  |  |
| *cox2i281g2* |  |  |  |  |  |  | + | + |  |  |
| ***cox2i373g2****** |  |  |  |  | + | + | + | + |  | *trans* |
| *cox2i564g2* |  |  |  |  |  |  | + |  |  |  |
| ***cox2i691g2***** |  |  |  |  | + | + | + |  |  |  |
| *cox3i109g2* |  |  |  |  |  |  | + |  |  |  |
| *cox3i171g2* |  | + | + | + |  |  |  |  |  |  |
| *cox3i506g2* |  |  |  |  | + | + |  |  |  |  |
| *cox3i625g2* |  | + | + | + |  |  |  |  |  |  |
| ***nad1i287g2***** |  |  |  |  | + | + | + | + |  |  |
| *nad1i348g2* |  |  |  |  |  |  | + | + |  |  |
| *nad1i394g2* |  |  |  |  |  |  |  |  | + | + |
| *nad1i477g2* |  |  |  |  |  |  |  |  |  | + |
| *nad1i669g2* |  |  |  |  |  |  |  |  |  | + |
| ***nad1i728g2****** |  |  |  |  | + | + | + | + |  | + |
| ***nad2i156g2***** |  |  |  |  | + | + |  |  | + | + |
| *nad2i542g2* |  |  |  |  |  |  |  |  | + | + |
| ***nad2i709g2****** |  | + | + | + |  |  | + | + | + | + |
| *nad2i830g2* |  |  |  |  |  |  |  |  | + | + |
| *nad2i1282g2* |  |  |  |  |  |  | + | + |  |  |
| ***nad3i52g2***** |  |  |  |  |  |  | + | + | + | + |
| ***nad3i140g2******* | + | + | + | + |  |  | + | + | + | + |
| *nad3i211g2* | + |  |  |  |  |  |  |  |  |  |
| ***nad4i461g2****** |  |  |  |  | + | + | + | + | + | + |
| *nad4i548g2* |  | + | + | + |  |  |  |  |  |  |
| ***nad4i976g2****** | + |  |  |  |  |  | + | + |  | + |
| *nad4i1399g2* |  |  |  |  |  |  |  |  | + | + |
| *nad4Li100g2* |  | + | + | + |  |  |  |  |  |  |
| ***nad4Li283g2***** |  |  | + | + | + | + |  |  |  |  |
| ***nad5i230g2***** |  |  |  |  | + | + | + | + |  |  |
| *nad5i242g2* |  |  |  |  |  |  |  |  | + | + |
| ***nad5i753g1***** |  | + | + | + | + | + |  |  |  |  |
| ***nad5i1455g2****** |  |  |  |  | + | + | + | + | + | + |
| ***nad5i1477g2***** |  |  |  |  |  |  | + | + | + | + |
| *nad6i444g2* |  |  |  |  |  |  | + | + |  |  |
| ***nad7i140g2***** |  |  |  |  | + | + |  |  |  | + |
| ***nad7i209g2***** |  |  |  |  | + | + |  |  | + | + |
| *nad7i336g2* |  | + | + | + |  |  |  |  |  |  |
| *nad7i676g2* |  |  |  |  |  |  |  |  | + | + |
| *nad7i917g2* |  |  |  |  |  |  |  |  | + | + |
| ***nad7i1113g2***** |  | + | + | + |  |  |  |  | + |  |
| *nad9i246g2* |  |  |  |  |  |  | + | + |  |  |
| *nad9i283g2* |  |  |  |  | + | + |  |  |  |  |
| *nad9i502g2* |  |  |  |  |  |  | + | + |  |  |
| *rpl2i28g2* |  | + | + | + |  |  |  |  |  |  |
| ***rps3i74g2***** | + |  |  |  |  |  |  |  | + |  |
| *rps14i114g2* |  | + | + | + |  |  |  |  |  |  |
| *rrn18i839g1* |  |  |  |  |  |  |  |  | + | + |
| *rrn18i1065g2* |  |  | + |  |  |  |  |  |  |  |
| *rrn26i819g1* | + |  |  |  |  |  |  |  |  |  |
| *rrn26i827g2* |  | + | + | + |  |  |  |  |  |  |
| *rrn26i1871g1* | + |  |  |  |  |  |  |  |  |  |
| *rrn26i1879g1* | + |  |  |  |  |  |  |  |  |  |
| *rrn26i1891g1* | + |  |  |  |  |  |  |  |  |  |
| *rrn26i2191g1* | + |  |  |  |  |  |  |  |  |  |
| *rrn26i2429g1* | + |  |  |  |  |  |  |  |  |  |
| *rrn26i2462g1* | + |  |  |  |  |  |  |  |  |  |
| *rrn26i2500g1* | + |  |  |  |  |  |  |  |  |  |
| *rrn26i2513g1* | + |  |  |  |  |  |  |  |  |  |
| ***sdh3i100g2***** |  |  |  |  | + | + | + | + |  |  |
| *trnNguui38g2* | + |  |  |  |  |  |  |  |  |  |
| *trnSgcui43g2* |  | + | + | + |  |  |  |  |  |  |

#### ^1^The full species names are as follows (in the order as they appear): *Chara vulgaris, Treubia lacunosa*, *Marchantia polymorpha, Pleurozia purpurea, Physcomitrella patens, Anomodon rugelii*, *Phaeoceros laevis, Megaceros aenigmaticus, Isoetes engelmannii,* and *Selaginella moellendorffii*. “+” indicates presence of an intron, and “*trans*” denotes a *trans*-spliced intron. Introns present in more than one clade are bold-faced, and the number of stars indicates the number of plants clades in which the intron is present. Intron nomenclature follows Dombrovska and Qiu 2004, and Knoop 2004.
